# The Association of Low-To-Moderate Alcohol Consumption with Breast Cancer Subtypes Defined by Hormone Receptor Status

**DOI:** 10.1371/journal.pone.0144680

**Published:** 2015-12-16

**Authors:** Loreta Strumylaite, Stephen J. Sharp, Rima Kregzdyte, Lina Poskiene, Algirdas Bogusevicius, Darius Pranys

**Affiliations:** 1 Neuroscience Institute, Medical Academy, Lithuanian University of Health Sciences, Kaunas, Lithuania; 2 MRC Epidemiology Unit, Institute of Metabolic Science, University of Cambridge School of Clinical Medicine, Cambridge, United Kingdom; 3 Department of Pathological Anatomy, Medical Academy, Lithuanian University of Health Sciences, Kaunas, Lithuania; 4 Department of Surgery, Medical Academy, Lithuanian University of Health Sciences, Kaunas, Lithuania; Wayne State University School of Medicine, UNITED STATES

## Abstract

**Background:**

Alcohol is a well-established risk factor for breast cancer, but pathways involved in alcohol*-*related breast carcinogenesis are not clearly defined. We examined the association between low-to-moderate alcohol intake and breast cancer subtypes by tumor hormone receptor status.

**Materials and Methods:**

A hospital-based case-control study was performed in 585 cases and 1,170 controls. Information on alcohol intake and other risk factors was collected via a questionnaire. Logistic regression was used for analyses. All statistical tests were two-sided.

**Results:**

The odds ratio of breast cancer was 1.75 (95% confidence interval [CI]: 1.21–2.53) in women who consumed ≤5 drinks/week, and 3.13 (95% CI: 1.81–5.43) in women who consumed >5 drinks/week, both compared with non-drinkers for ≥10 years, after adjustment for age and other confounders. The association of alcohol intake with estrogen receptor-positive breast cancer was stronger than with estrogen receptor-negative: the odds ratio per 1 category increase was 2.05 (95% CI: 1.49–2.82) and 1.29 (95% CI: 0.85–1.94) (P-heterogeneity = 0.07). There was no evidence of an interaction between alcohol intake and menopausal status (P = 0.19) in overall group; however, it was significant in estrogen receptor-positive breast cancer (P = 0.04).

**Conclusions:**

Low-to-moderate alcohol intake is associated with the risk of estrogen receptor-positive breast cancer with the strongest association in postmenopausal women. Since alcohol intake is a modifiable risk factor of breast cancer, every woman should be informed and advised to control alcohol use.

## Introduction

Many epidemiological studies have investigated the relationship between alcohol consumption and breast cancer risk [1]. An increased risk of breast cancer related to higher intake of alcohol has been found in most of the studies [2–5]. However, the findings on the association with low-to-moderate alcohol consumption are not consistent [6–10]. Little is known whether the association varies by menopausal status or other risk factors [6].

The underlying biological mechanism of the relationship between alcohol intake and breast cancer is not clearly defined [11]. However, there is some evidence that alcohol associated breast carcinogenesis includes hormone-dependent carcinogenic pathways. Ethanol stimulates cell proliferation and the transcriptional activity of estrogen receptor-α (ER-α), which enhances levels of circulating estrogens that control proliferation and morphogenesis in the breast [12–17]. Epidemiological studies also demonstrate evidence for endogenous sex hormones related increase in breast cancer risk [18, 19].

A significant increase in risk of estrogen receptor-positive (ER+) and/or estrogen receptor- and progesterone receptor-positive (ER+/PR+) breast cancer due to alcohol intake has been reported, but no increase in risk of estrogen receptor-negative (ER-) and/or estrogen receptor- and progesterone receptor-negative (ER-/PR-) breast cancer [3–5, 20–23]. However, the findings are not consistent [24–26]. Some authors identified a significant increase in risk of ER-/PR- [24, 25], but not in estrogen receptor-positive and progesterone receptor-negative (ER+/PR-) breast cancer [5, 20–22, 24].

This case-control study aimed to explore the association of low-to-moderate levels of alcohol consumption with risk of breast cancer subtypes defined by tumor hormone receptor status.

## Materials and Methods

### Study design

We performed a hospital-based case–control study of breast cancer in the Hospital of Lithuanian University of Health Sciences. The cases (n = 585, presenting 86.9% of eligible cases) were women aged 28–90 years with new histologically confirmed breast cancer (C50 and D05 according to ICD10) diagnosed between 1 March 2007 and 10 January 2011, who required surgical intervention at the Department of Surgery and were free from other cancer diagnosed in the past. The controls (n = 1,170, presenting 84.1% of eligible women) were women without a personal history of cancer hospitalized to other departments (Ophthalmology, Otolaryngology, Neurology, and Cardiology) of the hospital within the study period. Controls presented with a wide spectrum of non-neoplastic disorders and diseases of (a) eye (cataract, glaucoma, optic neuritis, and keratitis), (b) ear-nose-throat (otitis, sinusitis, deviation of nasal septum, tonsillitis), (c) nervous (facial and trigeminal neuritis, radiculopathy and radiculitis, epilepsia, multiple sclerosis, Parkinsonism, sleep disorders, and migraine), and (d) cardiovascular (arterial hypertension, ischemic disease, cardiomyopathia, different arrhythmias) systems. Controls were individually matched to cases by age (±5 years) in a 2:1 ratio. The study protocol was approved by the Kaunas Regional Biomedical Research Ethics Committee (10-01-2007 No. BE-2-1, Report No. 5/2007). Written consent to complete the questionnaire and collect biological media specimens was received from each individual.

### Questionnaire and exposure assessment

Both cases and controls completed a self-administered structured questionnaire previously demonstrated to be valid and reliable for collection of demographic and socioeconomic characteristics, medical history, height and weight, family history of cancer, reproductive history, and lifestyle characteristics [27].

Alcohol consumption was assessed by a question about the frequency/quantity of the use of three different types of alcoholic beverages such as spirits (vodka, brandy, liquor, and etc.), wine, and beer. The frequency of consumption was assigned to one of seven categories of never, once per two months, 1–3 times a month, once a week, 2–3 times a week, 4–6 times a week, every day. We also asked about quantities for each type of alcoholic beverage consumed in milliliters (ml). Assessment of alcohol consumption was based on an individual’s usual drinking habits. A recall period was set at one year before cancer diagnosis (cases) or the last admission to hospital (controls). We also ascertained alcohol consumption habits by the responses: “yes”, “gave up… years or… months ago”, “no, I have never used” to a question “Do/did you drink alcoholic beverages?” Therefore, if there was any recent change in habits or quitting drinking, information was sought on the respondent’s habits before the change.

The total number of alcoholic drinks (1 drink is about 10 g [0.01 kg] of alcohol) per week was calculated as the sum of the weekly drinks of three different types of alcoholic beverages (spirits, wine, and beer), each of which was equal to drinking frequency per week multiplied by quantity of specific type of alcohol, and divided by 32 ml [0.032 m^3^] for spirits or 120 ml [0.12 m^3^] for wine or 250 ml [0.25 m^3^] for beer, i.e. an amount of a specific alcohol type that contains about 10 g [0.01 kg] of alcohol [28]. Calculation of frequency scores was based on alcohol consumption per week. The scores were assigned as 0 for not users, 0.125 for once per two months (1/8 weeks), 0.5 for 1–3 times a month (2/4 weeks), 1 for once a week, 2.5 for 2–3 times per week, 5 for 4–6 days per week and 7 for every day.

### Measurements of hormone receptors

The estrogen receptor (ER) and progesterone receptor (PR) levels were measured in specimens of breast tumor tissue by immunohistochemistry at the Department of Pathological Anatomy [29].

### Statistical analysis

Baseline characteristics of cases and controls were summarized using means and standard deviations (SD) for continuous variables, and frequencies and percentages for categorical variables. Characteristics were compared between cases and controls using either unpaired t-tests (for continuous variables) or chi-squared tests (for categorical variables).

Women were grouped into 3 categories defined by the total number of alcoholic drinks consumed per week: 0 drinks for ≥10 years, ≤5 drinks/week, and >5 drinks/week. In the subsequent analyses, estimates of association per 1 category increase in alcohol intake were obtained; for some analyses estimates for ≤5 drinks/week vs. 0 drinks for ≥10 years, and >5 drinks/week vs. 0 drinks for ≥10 years were also reported.

According to tumor hormone receptor status cases were stratified as follows: ER+, ER-, progesterone receptor-positive (PR+), progesterone receptor-negative (PR-), ER+/PR+, ER+/PR-, ER-/PR+, and ER-/PR- [30].

Unconditional logistic regression models were used to estimate the association between alcohol intake and breast cancer subtypes calculating the odds ratios (ORs) and their 95% confidence intervals (CIs). Models were adjusted for (a) age and (b) age, number of births, age at first birth, estrogen-active (fertile) period, hormone therapy during menopause (never, estrogens and/or estrogens-progestin, other), family history of breast cancer in first and/or second degree of relatives (no, yes, unknown), smoking (never, ex-, current), body mass index, education (specialized secondary or lower, some university or higher), marital status (single, married or living as married, separated or widowed), diabetes mellitus (absent, present), and thyroid diseases (absent, present).

The interaction between menopausal status and alcohol intake (per 1 category increase) was tested using a likelihood ratio test. Heterogeneity in the estimated associations of alcohol with each breast cancer subtype was tested using a Cochran Q-test. The level of statistical significance was set at 0.05. All statistical tests were two-sided. The analyses were performed using software package Stata 10 (StataCorp LP, 2007).

## Results

Of the cases, 78.3% had invasive ductal carcinoma, 8.7% invasive lobular carcinoma, 13% other histological types of breast cancer. The ER+ and PR+ were determined for 65.3% and 45% of the cases (Table 1). The ER and PR levels were not measured for the cases with ductal and lobular carcinomas in situ (2.6%), and myoepithelial carcinoma (0.2%).

**Table 1:** Characteristics of breast cancer cases and controls.

Variable	Cases (n = 585)	Controls (n = 1,170)	P-value for difference
**Age (years) (mean, SD)**	58.19 (12.35)	57.42 (12.49)	0.22
Education (n, %)			
Specialized secondary or lower	358 (61.2)	816 (69.7)	
Some university or higher	227 (38.8)	354 (30.3)	<0.001
**Marital status (n, %)**			
Single	30 (5.1)	55 (4.7)	
Married or living as married	350 (59.8)	712 (60.9)	
Separated or widowed	205 (35.1)	403 (34.4)	0.88
**Family history of breast cancer (n, %)**	37 (6.3)	58 (5)	0.23
**Age at menarche (years) (mean, SD)**	14.05 (1.71)	14.01 (1.69)	0.67
**Age at first birth (years) (n, %)**			
<20	79 (13.5)	190 (16.2)	
20–29	412 (70.4)	830 (70.9)	
≥30	39 (6.7)	65 (5.6)	
Never gave birth	55 (9.4)	85 (7.3)	0.18
**Number of births (mean, SD)**	1.78 (1.1)	1.92 (1.08)	0.01
**Menopausal status (n, %)**			
Premenopausal	177 (30.3)	347 (29.7)	
Postmenopausal	408 (69.7)	823 (70.3)	0.74
**Estrogen-active (fertile) period (years)(mean, SD)** ^a^	34.32 (6.01)	33.21 (6.34)	<0.001
**Hormone therapy during menopause (n, %)**			
Never	335 (82.1)	725 (88.1)	
Estrogens and/or estrogens-progestin	50 (12.3)	71 (8.6)	
Other hormones (thyroxin and etc.)	23 (5.6)	27 (3.3)	0.01
**Alcohol use (n, %)**			
Never	24 (4.1)	90 (7.7)	
Ex-user	58 (9.9)	180 (15.4)	
Current	503 (86)	900 (76.9)	<0.001
**Alcohol intake (drinks/week)(n, %)**			
0 ^b^	43 (7.3)	146 (12.5)	
≤ 5	493 (84.3)	967 (82.6)	
> 5	49 (8.4)	57 (4.9)	<0.001
**Alcohol intake (drinks/week)(mean, SD)** ^c^	1.88 (4.37)	1.33 (3.04)	0.002
**Smoking (n, %)**			
Never	449 (76.8)	930 (79.5)	
Ex-smokers	61 (10.4)	127 (10.8)	
Current (every day or sometimes)	75 (12.8)	113 (9.7)	0.13
**Body mass index (kg/m** ^**2**^ **) (mean, SD)**	28.1 (5.63)	28.54 (5.94)	0.14
**Diabetes mellitus (n, %)**	37 (6.3)	118 (10.1)	0.01
**Thyroid diseases (n, %)**	127 (21.7)	317 (27.1)	0.01
**Tumor hormone receptors (n, %)** ^d^			
Estrogen receptor-positive	382 (65.3)	-	
Progesterone receptor-positive	263 (45)	-	

Abbreviations: SD, standard deviation.

^a^ Estrogen-active (fertile) period (years) = current age for non-menopausal women or age at menopause for postmenopausal women (years) minus age at menarche (years).

^b^ Non-drinkers for ≥10 years (reference group), i.e. never users (24 cases and 90 controls) and ex-drinkers who have stopped alcohol consumption ≥10 years ago (19 cases and 56 controls).

^c^ Calculation of mean of drinks/week is based on alcohol intake of all cases and controls.

^d^ The estrogen and progesterone receptors were not measured for the cases with ductal and lobular carcinomas in situ (n = 15, 2.6%), and myoepithelial carcinoma (n = 1, 0.2%).

Cases and controls were white Caucasians, similar with respect to marital status, family history of breast cancer, age at menarche and first birth, menopausal status, smoking, and body mass index (Table 1). However, cases had higher education and a longer estrogen-active (fertile) period, gave birth fewer times, and more often used hormone therapy during the menopause. But diabetes mellitus and thyroid diseases were more prevalent among controls.

Alcohol intake was greater among cases than controls. Current alcohol users were 86% of cases and 76.9% of controls (P < 0.001) (Table 1). However, both cases and controls had a low mean alcohol intake: 1.88 (SD = 4.37) drinks/week for cases and 1.33 (SD = 3.04) drinks/week for controls (P = 0.002).

A significant increase in the odds of breast cancer associated with increased alcohol intake was seen in the adjusted model (Table 2). Compared with non-drinkers for ≥10 years, the OR for consumers of ≤5 drinks/week was 1.75 (95% CI: 1.21–2.53) and for consumers of >5 drinks/week was 3.13 (95% CI: 1.81–5.43; P-trend < 0.001). When stratified by menopausal status, the association appeared stronger among postmenopausal women, but the interaction between menopausal status and alcohol intake was not significant (P = 0.19) (Table 2).

**Table 2:** Association between alcohol intake and breast cancer.

Alcohol intake(drinks/week)	Cases	Controls	OR (95% CI) ^a^	OR (95% CI) ^b^
	n (%)	n (%)		
**All women**	585	1170		
0 ^c^	43 (7.3)	146 (12.5)	1.0	1.0
≤ 5	493 (84.3)	967 (82.6)	1.83 (1.28–2.62)	1.75 (1.21–2.53)
> 5	49 (8.4)	57 (4.9)	3.39 (2.0–5.74)	3.13 (1.81–5.43)
OR per 1 category increase			1.84 (1.41–2.39)	1.77 (1.35–2.33)
P _trend_			<0.001	<0.001
P _interaction_ ^†^			0.23	0.19
**Postmenopausal women**	407	823		
0 ^c^	38 (9.4)	124 (15.1)	1.0	1.0
≤ 5	344 (84.5)	681 (82.7)	1.7 (1.15–2.51)	1.7 (1.14–2.53)
> 5	25 (6.1)	18 (2.2)	4.96 (2.4–10.17)	5.04 (2.4–10.64)
OR per 1 category increase			2.0 (1.44–2.77)	1.98 (1.42–2.78)
P _trend_			<0.001	<0.001
**Premenopausal women**	178	347		
0 ^c^	5 (2.8)	22 (6.4)	1.0	1.0
≤ 5	149 (83.7)	286 (82.4)	2.5 (0.92–6.78)	2.19 (0.78–6.16)
> 5	24 (13.5)	39 (11.2)	3.31 (1.1–10.06)	2.57 (0.8–8.27)
OR per 1 category increase			1.57 (1.0–2.48)	1.38 (0.85–2.24)
P _trend_			0.05	0.19

Abbreviations: OR, odds ratio; CI, confidence interval.

^a^ Adjusted for age.

^b^ Further adjustment for number of births, age at first birth, estrogen-active (fertile) period, hormone therapy during menopause, family history of breast cancer, smoking, body mass index, education, marital status, diabetes mellitus, and thyroid diseases.

^c^ Non-drinkers for ≥10 years (reference group).

^†^ P-value from likelihood ratio test of interaction between menopausal status and alcohol intake (per 1 category increase).

After adjustment for age and other confounders, a significant positive association between alcohol and ER+ breast cancer was identified: the OR per 1 alcohol category increase was 2.05 (95% CI: 1.49–2.82; P-trend < 0.001) (Table 3).

**Table 3:** Association between alcohol intake and breast cancer defined by estrogen receptor and progesterone receptor status. ^¶^

Alcohol intake (drinks/week)	n controls	ER+		ER-		P_heter_ ^†^	PR+		PR-		P_heter_ ^†^
		n cases	OR(95% CI) ^a^	n cases	OR(95% CI) ^a^		n cases	OR (95% CI) ^a^	n cases	OR (95% CI) ^a^	
**All women**	1170	382		187			263		306		
0 ^b^	146	24	1.0	18	1.0		16	1.0	26	1.0	
≤ 5	967	327	2.29 (1.44–3.64)	154	1.12 (0.66–1.92)		226	2.15 (1.25–3.74)	255	1.51 (0.96–2.39)	
> 5	57	31	4.16 (2.15–8.05)	15	1.71 (0.76–3.83)		21	3.16 (1.48–6.77)	25	3.01 (1.52–5.93)	
OR per 1 category increase			2.05 (1.49–2.82)		1.29 (0.85–1.94)			1.79 (1.24–2.57)		1.7 (1.21–2.4)	
P_trend_			<0.001		0.23	0.07		0.002		0.002	0.85
P_interaction_ ^‡^			0.04		0.98			0.05		0.51	
**Postmenopausal women**	823	277		120			170		227		
0 ^b^	124	21	1.0	16	1.0		14	1.0	23	1.0	
≤ 5	681	238	2.32 (1.4–3.84)	98	1.02 (0.57–1.81)		145	2.0 (1.1–3.63)	191	1.51 (0.93–2.46)	
> 5	18	18	7.41 (3.17–17.33)	6	2.55 (0.83–7.85)		11	6.25 (2.3–16.73)	13	4.52 (1.85–11.09)	
OR per 1 category increase			2.53 (1.69–3.81)		1.27 (0.75–2.15)			2.32 (1.43–3.77)		1.81 (1.19–2.77)	
P_trend_			<0.001		0.37	0.04		0.001		0.006	0.45
**Premenopausal women**	347	105		67			93		79		
0 ^b^	22	3	1.0	2	1.0		2	1.0	3	1.0	
≤ 5	286	89	2.17 (0.6–7.83)	56	2.2 (0.47–10.36)		81	2.87 (0.63–13.1)	64	1.72 (0.47–6.24)	
> 5	39	13	2.05 (0.47–8.82)	9	2.47 (0.44–13.78)		10	2.08 (0.39–11.26)	12	2.48 (0.57–10.7)	
OR per 1 category increase			1.26 (0.7–2.24)		1.22 (0.62–2.4)			1.08 (0.59–1.99)		1.42 (0.76–2.65)	
P_trend_			0.44		0.56	0.95		0.79		0.27	0.54

Abbreviations: OR, odds ratio; CI, confidence interval; ER+, estrogen receptor-positive; ER-, estrogen receptor-negative; PR+, progesterone receptor-positive; PR-, progesterone receptor-negative.

^¶^ Analysis included women with determined tumor hormone receptors (n = 569).

^a^ Adjusted for age, number of births, age at first birth, estrogen-active (fertile) period, hormone therapy during menopause, family history of breast cancer, smoking, body mass index, education, marital status, diabetes mellitus, and thyroid diseases.

^b^ Non-drinkers for ≥10 years (reference group).

^†^ P—value from Cochran Q test of heterogeneity in the associations between alcohol intake (per 1 category increase) and (1) ER+ or ER- breast cancer, (2) PR+ or PR- breast cancer.

^‡^ P—value from likelihood ratio test of interaction between menopausal status and alcohol intake (per 1 category increase).

There was no evidence of an association between alcohol and ER- breast cancer (P-trend = 0.23). The alcohol/ER+ breast cancer association appeared stronger among postmenopausal women than premenopausal women (P-interaction = 0.04).

Significant positive associations between alcohol and both PR+ and PR- breast cancer were found in overall and in postmenopausal women, with no evidence of heterogeneity in the association comparing PR+ and PR- breast cancer (P > 0.05), but with a suggestion of an interaction between menopausal status and alcohol intake for PR+ breast cancer (P = 0.05) (Table 3).

There were significant positive associations between alcohol and (1) ER+/PR-, (2) ER+/PR+ breast cancer, but not for ER-/PR- breast cancer (Table 4). A 1 category increase in alcohol intake was associated with an OR of 2.57 (95% CI: 1.53–4.3; P-trend < 0.001) for ER+/PR- breast cancer, which was significantly different from the alcohol/ER-/PR- association (P-heterogeneity = 0.04), and with an OR of 1.81 (95% CI: 1.25–2.62; P-trend = 0.002) for ER+/PR+ breast cancer, not significantly different from the alcohol/ER-/PR- association (P-heterogeneity = 0.24). Significant positive associations appeared stronger in postmenopausal women compared with premenopausal women; however, the 95% CIs for the ORs in premenopausal women overlap with those for postmenopausal women, and the P-interaction values were not significant for either breast cancer subtype (Table 4).

**Table 4:** Association between alcohol intake and breast cancer defined by joint hormone receptor status.^¶^

Alcohol intake (drinks/week)	n controls	ER-/PR-		ER+/PR-		ER+/PR+		ER-/PR+	
		n cases	OR (95% CI) ^a^	n cases	OR (95% CI)^a^	n cases	OR (95% CI)^a^	n cases	OR (95% CI) ^a^
**All women**	1170	174		132		250		13	
0 ^b^	146	18	1.0	8	1.0	16	1.0	0	-
≤ 5	967	142	1.07 (0.63–1.84)	113	2.72 (1.26–5.84)	214	2.09 (1.21–3.61)	12	-
> 5	57	14	1.77 (0.78–4.01)	11	6.73 (2.37–19.07)	20	3.27 (1.52–7.05)	1	-
OR per 1 category increase			1.29 (0.85–1.97)		2.57 (1.53–4.3)		1.81 (1.25–2.62)		1.53 (0.37–6.31)
P_trend_			0.23		<0.001		0.002		0.56
P_heterogeneity_ ^†^			-		0.04		0.24		0.83
P_interaction_ ^‡^			0.66		0.13		0.12		-
**Postmenopausal women**	823	116		111		166		4	
0 ^b^	124	16	1.0	7	1.0	14	1.0	0	-
≤ 5	681	95	1.0 (0.56–1.78)	96	2.92 (1.29–6.63)	142	1.97 (1.08–3.57)	3	-
> 5	18	5	2.24 (0.69–7.26)	8	11.07 (3.28–37.4)	10	5.91 (2.17–16.08)	1	-
OR per 1 category increase			1.2 (0.71–2.04)		3.14 (1.69–5.83)		2.24 (1.37–3.65)		-
P_trend_			0.49		<0.001		0.001		
P_heterogeneity_ ^†^			-		0.02		0.09		
**Premenopausal women**	347	58		21		84		9	
0 ^b^	22	2	1.0	1	1.0	2	1.0	0	-
≤ 5	286	47	1.92 (0.41–9.04)	17	1.08 (0.12–9.51)	72	2.6 (0.57–11.86)	9	-
> 5	39	9	2.82 (0.51–15.67)	3	1.05 (0.08–14.3)	10	2.33 (0.43–12.64)	0	-
OR per 1 category increase			1.41 (0.7–2.84)		1.32 (0.43–4.01)		1.19 (0.64–2.22)		0.55 (0.07–4.22)
P_trend_			0.34		0.63		0.59		0.57
P_heterogeneity_ ^†^			-		0.92		0.72		0.82

Abbreviations: OR, odds ratio; CI, confidence interval; ER-/PR-, estrogen receptor-negative and progesterone receptor-negative; ER+/PR-, estrogen receptor-positive and progesterone receptor-negative; ER+/PR+, estrogen receptor-positive and progesterone receptor-positive; ER-/PR+, estrogen receptor-negative and progesterone receptor-positive.

^¶^ Analysis included women with determined tumor hormone receptors (n = 569).

^a^ Adjusted for age, number of births, age at first birth, estrogen-active (fertile) period, hormone therapy during menopause, family history of breast cancer, smoking, body mass index, education, marital status, diabetes mellitus, and thyroid diseases.

^b^ Non-drinkers for ≥10 years (reference group).

^**†**^ P—value from Cochran Q test of heterogeneity in the associations between alcohol intake (per 1 category increase) and either ER-/PR- or one of the following ER+/PR-, ER+/PR+, and ER-/PR+ breast cancer.

^**‡**^ P—value from likelihood ratio test of interaction between menopausal status and alcohol intake (per 1 category increase).

## Discussion

In this case–control study, in which cases and controls consumed on average 1.88 and 1.33 drinks/week, we identified an association between low-to-moderate alcohol intake and risk of breast cancer: 1 category increase in alcohol intake was associated with a 1.8-fold increase in the odds of breast cancer. The findings are in agreement with results presented by most other studies [2–6, 8, 31, 32], although some studies, especially those which estimated low-to-moderate alcohol intake related risk to breast cancer, did not find an association [7, 9, 10].

We found significant alcohol/breast cancer associations for all ER+ (ER+, ER+/PR+, and ER+/PR-), but not for ER- (ER- and ER-/PR-) subtypes. The findings are in line with a meta-analysis which demonstrated an increased risk of ER+ breast cancer, and no association between alcohol and ER-/PR- breast cancer [3]. A greater risk of ER+ breast cancer related to increased alcohol intake was reported by other authors as well [3–5, 20–23, 33] but the findings are not consistent [25, 26]. Two cohort studies published recently observed alcohol/breast cancer association independent of ER/PR status [32, 34].

Increased alcohol intake was associated with higher risk of both PR+ and PR- breast cancer, with no evidence of heterogeneity in the association. The last finding, together with the facts that (a) in this study 95% of PR+ and 43% of PR- breast cancer were also ER+ breast cancer; (b) a significant association defined for ER+, but not for ER- breast cancer with potential heterogeneity (P = 0.07) in overall group and significant heterogeneity (P = 0.04) in postmenopausal women, leads us to believe that ER status may explain the significant association of alcohol with PR+ breast cancer and, in part, with PR- breast cancer. Our findings as well as results from other epidemiological studies [3–5, 20–23, 33] support the importance of an estrogen-dependent pathway in alcohol induced breast carcinogenesis. The results are corroborated by experimental studies. Enhanced sex hormone levels after intake of moderate levels of alcohol were found in both premenopausal and postmenopausal women [14, 15]. There is evidence that endogenous estrogen levels are increased by alcohol because of decreased hepatic catabolism of androgens [35] or effects on adrenal steroid production [15]. In vitro studies demonstrate that alcohol stimulates proliferation, ER-α and aromatase expression in breast cancer cells [36], increases the transcriptional activity of ER-α [12] and enhances the content of ER-α in ER+ human breast cancer cells [13].

Our rationale for assessing potential heterogeneity in the alcohol/breast cancer association by menopausal status was firstly, because most of breast cancers are postmenopausal with the majority being hormone dependent [37]; secondly, alcohol (ethanol) increases content of estrogens that are associated with higher risk of breast cancer [14, 15, 18, 19]. In the overall sample, we did not find a significant interaction between alcohol and menopausal status; however, when considering different breast cancer subtypes defined by hormone receptors, there was a significant interaction between menopausal status and alcohol for hormone receptors-positive (ER+ or PR+) breast cancer, and no evidence of interaction for hormone receptors-negative (ER- or PR-) breast cancer. Again, knowing that in this study 98% of postmenopausal PR+ breast cancer was also ER+ breast cancer, postmenopausal ER+ women seemed to be at greater risk of breast cancer due to alcohol intake. This assumption is supported by the results on the association between alcohol intake and breast cancer defined by joint hormone receptor status. However, the small number of premenopausal cases with certain tumor receptors meant there was considerable uncertainty about the true magnitude of any associations for these groups. Yet, our results add further support to the importance of hormonal mechanisms in alcohol induced breast carcinogenesis and stress its possible differences due to menopausal status in hormone receptor-positive breast cancer. Epidemiological findings on alcohol/breast cancer association concerning the menopausal status are not consistent [8, 10, 26, 38]. A meta-analysis of epidemiological studies did not find different associations by menopausal status [39], however, interaction between menopausal status and alcohol in breast cancer subtypes defined by hormone receptors was not explored.

In this study, in which controls were individually matched to cases by age, to estimate the association we used unconditional logistic regression (adjusted for age and other risk factors) rather than conditional logistic regression. Conditional logistic regression was more powerful and showed stronger effects in the overall sample; however, when considering different breast cancer subtypes defined by hormone receptors and, especially by menopausal status, the small number of individuals with certain breast cancer subtypes led to wide CIs around the estimated associations, especially for premenopausal women.

To avoid selection bias, we invited all the patients that met selection criteria within the study period at the departments of the hospital selected randomly and did not disclose the hypothesis. Both cases and controls were representative of patients in a hospital which provides medical services for the population. This type of the study ensured a high response rate in both cases and controls; 13.1% of cases and 15.9% of controls declined to participate in the study (P = 0.09).

We assessed exposure to alcohol by number of drinks per week, where one drink was equivalent to 10 g [0.01 kg] of pure ethanol that was in 32 ml [0.032 m^3^] of spirits or 120 ml [0.12 m^3^] of wine or 250 ml [0.25 m^3^] of beer [28]. However, ethanol quantities may vary in different alcoholic beverages and a real quantity of pure ethanol in one drink may differ from that was assumed [40]. Nevertheless, we tried to be as precise as possible, asking about both the frequency and amount consumed, for beer, wine, and liquor, separately [41], for the recall period that was a year before cancer diagnosis (cases) or the last admission to hospital (controls). We also took into account recent change in habits or quitting drinking, and information was sought on the respondent’s habits before the change. However, a history of alcohol use with possibly previous changes was not assessed. On the one hand, this “reference” recall period gave more accurate information because of lower recall bias, a known limitation of case-control studies. On the other hand, some information about previous alcohol use and possible changes were not incorporated into the analysis.

In conclusion, this study provides evidence that the association between low-to-moderate alcohol intake and risk of breast cancer differs by tumor hormone receptor status, with a stronger risk demonstrated for estrogen receptor-positive breast cancer. The results show that alcohol/estrogen receptor-positive breast cancer association differs by menopausal status. Further studies are necessary to define whether the association with alcohol consumption varies by menopausal status independently of other risk factors. Since alcohol intake is a modifiable risk factor of breast cancer, the findings are very important to public health and primary health care workers that could inform and advise women to control alcohol consumption.

## Supporting Information

S1 Data Data BC alcohol. (DTA)

## References

[1] IARC (International Agency for Research on Cancer). Alcohol consumption and ethyl carbamate IARC monographs on the evaluation of carcinogenic risks to humans. Lyon: IARC; 2010. PMC4781168 21735939

[2] Hamajima N, Hirose K, Tajima K, Rohan T, Calle EE, Heath CW Jr, et al Alcohol, tobacco and breast cancer—collaborative reanalysis of individual data from 53 epidemiological studies, including 58,515 women with breast cancer and 95,067 women without the disease. Br J Cancer. 2002; 87: 1234–1245. 12439712 10.1038/sj.bjc.6600596 PMC2562507

[3] Suzuki R, Orsini N, Mignone L, Saji S, Wolk A. Alcohol intake and risk of breast cancer defined by estrogen and progesterone receptor status—a meta-analysis of epidemiological studies. Int J Cancer. 2008; 122: 1832–1841. 18067133 10.1002/ijc.23184

[4] Li CI, Chlebowski RT, Freiberg M, Johnson KC, Kuller L, Lane D, et al Alcohol consumption and risk of postmenopausal breast cancer by subtype: the women's health initiative observational study. J Natl Cancer Inst. 2010; 102: 1422–1431. 10.1093/jnci/djq316 20733117 PMC2943525

[5] Zhang SM, Lee IM, Manson JE, Cook NR, Willett WC, Buring JE. Alcohol consumption and breast cancer risk in the Women’s Health Study. Am J Epidemiol. 2007; 165: 667–676. 17204515 10.1093/aje/kwk054

[6] Allen NE, Beral V, Casabonne D, Kan SW, Reeves GK, Brown A, et al Moderate alcohol intake and cancer incidence in women. J Natl Cancer Inst. 2009; 101: 296–305. 10.1093/jnci/djn514 19244173

[7] Zhang M, Holman CDJ. Low-to-moderate alcohol intake and breast cancer risk in Chinese women. Br J Cancer. 2011; 105: 1089–1095. 10.1038/bjc.2011.302 21829196 PMC3185931

[8] Chen WY, Rosner B, Hankinson SE, Colditz GA, Willett WC. Moderate alcohol consumption during adult life, drinking patterns, and breast cancer risk. JAMA. 2011; 306: 1884–1990. 10.1001/jama.2011.1590 22045766 PMC3292347

[9] Brown LM, Gridley G, Wu AH, Falk RT, Hauptmann M, Kolonel LN, et al Low level alcohol intake, cigarette smoking and risk of breast cancer in Asian American women. Breast Cancer Res Treat. 2010; 120: 203–210. 10.1007/s10549-009-0464-4 19597702 PMC2808456

[10] Kropp S, Becher H, Nieters A, Chang-Claude J. Low-to-moderate alcohol consumption and breast cancer risk by age 50 years among women in Germany. Am J Epidemiol. 2001; 154: 624–634. 11581096 10.1093/aje/154.7.624

[11] Visvanathan K, Crum RM, Strickland PT, You X, Ruczinski I, Berndt SI, et al Alcohol dehydrogenase genetic polymorphisms, low-to-moderate alcohol consumption, and risk of breast cancer. Alcohol Clin Exp Res. 2007; 31: 467–476. 17295732 10.1111/j.1530-0277.2006.00334.x PMC2787101

[12] Fan S, Meng Q, Gao B, Grossman J, Yadegari M, Goldberg ID, et al Alcohol stimulates estrogen receptor signaling in human breast cancer cell lines. Cancer Res. 2000; 60: 5635–5639. 11059753

[13] Singletary KW, Frey RS, Yan W. Effect of ethanol on proliferation and estrogen receptor-alpha expression in human breast cancer cells. Cancer Lett. 2001; 165: 131–137. 11275361 10.1016/s0304-3835(01)00419-0

[14] Reichman ME, Judd JT, Longcope C, Schatzkin A, Clevidence BA, Nair PP, et al Effects of alcohol consumption on plasma and urinary hormone concentrations in premenopausal women. J Natl Cancer Inst. 1993; 85: 722–727. 8478958 10.1093/jnci/85.9.722

[15] Dorgan JF, Baer DJ, Albert PS, Judd JT, Brown ED, Corle DK, et al Serum hormones and the alcohol-breast cancer association in postmenopausal women. J Natl Cancer Inst. 2001; 93: 710–715. 11333294 10.1093/jnci/93.9.710

[16] Gavaler JS, Rosenblum E. Exposure dependent effects of ethanol on serum estradiol and uterus mass in sexually mature oophorectomized rats: a model for bilaterally ovariectomized-postmenopausal women. J Stud Alcohol. 1987; 48: 295–303. 3613579 10.15288/jsa.1987.48.295

[17] Ginsburg ES, Walsh BW, Shea BF, Gao X, Gleason RE, Barbieri RL. The effects of ethanol on the clearance of estradiol in postmenopausal women. Fertil Steril. 1995; 63: 1227–1230. 7750592

[18] Key T, Appleby P, Barnes I, Reeves G; Endogenous Hormones and Breast Cancer Collaborative Group. Endogenous sex hormones and breast cancer in postmenopausal women: reanalysis of nine prospective studies. J Natl Cancer Inst. 2002; 94: 606–616. 11959894 10.1093/jnci/94.8.606

[19] Dallal CM, Tice JA, Buist DS, Bauer DC, Lacey JV Jr, Cauley JA, et al Estrogen metabolism and breast cancer risk among postmenopausal women: a case-cohort study within B~FIT. Carcinogenesis. 2014; 35: 346–355. 10.1093/carcin/bgt367 24213602 PMC3908751

[20] Li Y, Baer D, Friedman GD, Udaltsova N, Shim V, Klatsky AL. Wine, liquor, beer and risk of breast cancer in a large population. Eur J Cancer. 2009; 45: 843–850. 10.1016/j.ejca.2008.11.001 19095438

[21] Deandrea S, Talamini R, Foschi R, Montella M, Dal Maso L, Falcini F, et al Alcohol and breast cancer risk defined by estrogen and progesterone receptor status: a case-control study. Cancer Epidemiol Biomarkers Prev. 2008; 17: 2025–2028. 10.1158/1055-9965.EPI-08-0157 18708394

[22] Lew JQ, Freedman ND, Leitzmann MF, Brinton LA, Hoover RN, Hollenbeck AR, et al Alcohol and risk of breast cancer by histologic type and hormone receptor status in postmenopausal women: the NIH-AARP Diet and Health Study. Am J Epidemiol. 2009; 170: 308–317. 10.1093/aje/kwp120 19541857 PMC2727171

[23] Suzuki R, Ye W, Rylander-Rudqvist T, Saji S, Colditz GA, Wolk A. Alcohol and postmenopausal breast cancer risk defined by estrogen and progesterone receptor status: a prospective cohort study. J Natl Cancer Inst. 2005; 97: 1601–1608. 16264180 10.1093/jnci/dji341

[24] Setiawan VW, Monroe KR, Wilkens LR, Kolonel LN, Pike MC, Henderson BE. Breast cancer risk factors defined by estrogen and progesterone receptor status: the multiethnic cohort study. Am J Epidemiol. 2009; 169: 1251–1259. 10.1093/aje/kwp036 19318616 PMC2727208

[25] Rosenberg LU, Einarsdóttir K, Friman EI, Wedrén S, Dickman PW, Hall P, et al Risk factors for hormone receptor-defined breast cancer in postmenopausal women. Cancer Epidemiol Biomarkers Prev. 2006; 15: 2482–2488. 17164374 10.1158/1055-9965.EPI-06-0489

[26] Suzuki R, Iwasaki M, Inoue M, Sasazuki S, Sawada N, Yamaji T, et al Alcohol consumption-associated breast cancer incidence and potential effect modifiers: the Japan Public Health Center-based Prospective Study. Int J Cancer. 2010; 127: 685–695. 10.1002/ijc.25079 19960437

[27] Strumylaite L, Kregzdyte R, Rugyte DC, Bogusevicius A, Mechonosina K. Assessment of a questionnaire for breast cancer case-control studies. Asian Pac J Cancer Prev. 2013; 14: 2777–2782. 23803031 10.7314/apjcp.2013.14.5.2777

[28] WHO (World Health Organization). International guide for monitoring alcohol consumption and related harm. Geneva: WHO; 2000.

[29] DAKO. Reference in Immunohistochemistry Breast cancer diagnosis, therapy and prognosis, 3rd ed 00079b/10000. Glostrup: DAKo A/S, 1996 p.9.

[30] Hammond ME, Hayes DF, Wolff AC, Mangu PB, Temin S. American society of clinical oncology/college of American pathologists guideline recommendations for immunohistochemical testing of estrogen and progesterone receptors in breast cancer. J Oncol Pract. 2010; 6: 195–197. 10.1200/JOP.777003 21037871 PMC2900870

[31] Cao Y, Willett WC, Rimm EB, Stampfer MJ, Giovannucci EL. Light to moderate intake of alcohol, drinking patterns, and risk of cancer: results from two prospective US cohort studies. BMJ. 2015 8 18; 351: h4238 10.1136/bmj.h4238 26286216 PMC4540790

[32] Park SY, Kolonel LN, Lim U, White KK, Henderson BE, Wilkens LR. Alcohol consumption and breast cancer risk among women from five ethnic groups with light to moderate intakes: the Multiethnic Cohort Study. Int J Cancer. 2014; 134: 1504–1510. 10.1002/ijc.28476 24037751 PMC4102309

[33] Kabat GC, Kim M, Phipps AI, Li CI, Messina CR, Wactawski-Wende J, et al Smoking and alcohol consumption in relation to risk of triple-negative breast cancer in a cohort of postmenopausal women. Cancer Causes Control. 2011; 22: 775–783. 10.1007/s10552-011-9750-7 21360045 PMC3100347

[34] Romieu I, Scoccianti C, Chajès V, de Batlle J, Biessy C, Dossus L, et al Alcohol intake and breast cancer in the European prospective investigation into cancer and nutrition. Int J Cancer. 2015; 137: 1921–1930. 10.1002/ijc.29469 25677034 PMC6300114

[35] Sarkola T, Adlercreutz H, Heinonen S, von Der Pahlen B, Eriksson CJ. The role of the liver in the acute effect of alcohol on androgens in women. J Clin Endocrinol Metab. 2001; 86: 1981–1985. 11344195 10.1210/jcem.86.5.7486

[36] Etique N, Chardard D, Chesnel A, Merlin JL, Flament S, Grillier-Vuissoz I. Ethanol stimulates proliferation, ER-α and aromatase expression in MCF-7 human breast cancer cells. Int J Mol Med. 2004; 13: 149–155. 14654987

[37] Miller TW, Hennessy BT, Gonzalez-Angulo AM, Fox EM, Mills GB, Chen H, et al Hyperactivation of phosphatidylinositol-3 kinase promotes escape from hormone dependence in estrogen receptor–positive human breast cancer. J Clin Invest. 2010; 120: 2406–2413. 10.1172/JCI41680 20530877 PMC2898598

[38] Friedenreich CM, Howe GR, Miller AB, Jain MG. A cohort study of alcohol consumption and risk of breast cancer. Am J Epidemiol. 1993; 137: 512–520. 8465803 10.1093/oxfordjournals.aje.a116704

[39] Key J, Hodgson S, Omar RZ, Jensen TK, Thompson SG, Boobis AR, et al Meta-analysis of studies of alcohol and breast cancer with consideration of the methodological issues. Cancer Causes Control. 2006; 17: 759–770. 16783604 10.1007/s10552-006-0011-0

[40] Secretan B, Straif K, Baan R, Grosse Y, El Ghissassi F, Bouvard V, et al A review of human carcinogens—Part E: tobacco, areca nut, alcohol, coal smoke, and salted fish. Lancet Oncol. 2009; 10: 1033–1034. 19891056 10.1016/s1470-2045(09)70326-2

[41] Feunekes GI, Veer P, Staveren WA, Kok FJ. Alcohol intake assessment: the sober facts. Am J Epidemiol. 1999; 150: 105–112. 10400547 10.1093/oxfordjournals.aje.a009909

